# Electromyography Frequency Spectrum Is a Better Indicator of Sleep Bruxism Severity Related to Temporomandibular Disorder Pain Than Conventional Masticatory Muscle Activation and Bruxism Time Indices—A Pilot Study

**DOI:** 10.1111/jsr.70287

**Published:** 2026-01-25

**Authors:** Minna Pitkänen, Miro Rytkönen, Tomi Miettinen, Jari Ahlberg, Frank Lobbezoo, Katja Myllymaa, Juha Töyräs, Timo Leppänen, Sami Myllymaa

**Affiliations:** ^1^ Department of Technical Physics University of Eastern Finland Kuopio Finland; ^2^ Diagnostic Imaging Centre, Kuopio University Hospital, Wellbeing Services County of North Savo Kuopio Finland; ^3^ Clinical Research Centre, Kuopio University Hospital, Wellbeing Services County of North Savo Kuopio Finland; ^4^ Comprehensive Cancer Center, Helsinki University Hospital and University of Helsinki Helsinki Finland; ^5^ Department of Oral and Maxillofacial Diseases University of Helsinki Helsinki Finland; ^6^ Head and Neck Center, Helsinki University Hospital Helsinki Finland; ^7^ Department of Orofacial Pain & Dysfunction Academic Centre for Dentistry Amsterdam (ACTA), University of Amsterdam and Vrije Universiteit Amsterdam Amsterdam the Netherlands; ^8^ Department of Orofacial Pain and Jaw Function Faculty of Odontology, Malmö University Malmö Sweden; ^9^ School of Electrical Engineering and Computer Science, The University of Queensland Brisbane Australia; ^10^ Science Service Center, Kuopio University Hospital, Wellbeing Services County of North Savo Kuopio Finland

**Keywords:** bruxism, electromyography, frequency spectrum, pain, temporomandibular disorders

## Abstract

Sleep bruxism (SB) has been reportedly associated with temporomandibular disorder (TMD); however, solid evidence is lacking. Previous studies have primarily used traditional metrics, such as the masticatory muscle activity (MMA) index and bruxism time index (BTI) to investigate the link between SB and TMD. However, we aimed to examine how the electromyography (EMG) frequency spectrum is associated with TMD in SB participants. We hypothesised that the EMG signal frequencies during MMA events would be lower in SB participants with TMD pain compared to those without TMD pain. In this exploratory study, we retrospectively analysed home polysomnography data from 44 participants who indicated possible SB. The median signal frequencies and absolute power were calculated using the Fast Fourier Transform of the EMG signals during MMA events. Moreover, the MMA index and BTI were calculated, and all parameters were compared between SB participants with and without TMD pain. The results showed that the absolute power and median frequencies were significantly lower in SB participants with TMD pain compared to those without TMD pain (*p* < 0.05), whereas the MMA index and BTI did not differ between the groups (*p* > 0.05). These findings suggest that masticatory muscles are getting fatigued in TMD participants with SB and therefore, EMG frequency‐based analysis may provide a promising direction for future assessment of TMD consequences of SB. However, these preliminary results should be validated in future studies involving a larger and more heterogeneous pool of participants.

## 1 Introduction

Sleep bruxism (SB) is defined as masticatory muscle activity (MMA) during sleep (Lobbezoo et al. 2018, 2013; Verhoeff et al. 2025). This activity, which can be rhythmic (phasic) or non‐rhythmic (tonic), is not considered a disorder in otherwise healthy individuals (Lobbezoo et al. 2018; Verhoeff et al. 2025) and is mainly regulated centrally rather than peripherally (Lobbezoo and Naeije 2001). SB can be classified into three grades: possible, probable, and definite (Lobbezoo et al. 2018, 2013). When graded as possible, bruxism is based on a positive self‐report only, while probable bruxism requires a positive clinical inspection (Lobbezoo et al. 2018). Definite bruxism is based on a positive instrumental assessment, such as electromyography (EMG) (Lobbezoo et al. 2018). Previously, it has been proposed that bruxism may be a risk factor for negative clinical consequences, such as temporomandibular disorder (TMD) (Svensson et al. 2008), but more recently it has been suggested that it may also have positive consequences, such as restoring airway patency during sleep (Lobbezoo et al. 2018, 2013).

TMD is a musculoskeletal disorder involving pain and tenderness in the temporomandibular joints, masticatory muscles, or associated tissues, and is often associated with limited jaw movements and temporomandibular joint sounds. The association between SB and TMD is controversial (Manfredini and Lobbezoo 2021; Lobbezoo and Lavigne 1997). Previously, TMD has been associated with both self‐reported SB (Velly et al. 2003; Fernandes et al. 2012) and instrumentally assessed SB (Rossetti, de Araujo, et al. 2008; Barbon et al. 2023; Martynowicz et al. 2024). However, several studies using polysomnography have not found a link between SB and TMD (Camparis et al. 2006; Rossetti, Rossetti, et al. 2008; Raphael et al. 2012; Wieckiewicz et al. 2025) and some have even found a negative correlation between SB and TMD pain (Arima et al. 2001; Lavigne et al. 1997; Van Der Zaag et al. 2002).

Most previous studies have focused solely on the number or duration of MMA events, and typically, the EMG signal is otherwise ignored. The frequency spectrum of the EMG yields different insights into muscle activity compared to time domain analysis; lower frequencies in the EMG signal reflect muscle fatigue (Naeije and Zorn 1981). Therefore, we aimed to investigate the EMG frequency spectrum during MMA events and compare it between SB participants with and without TMD pain (SB + TMD and SB − TMD, respectively). We hypothesised that masticatory muscles become more fatigued in SB + TMD compared to SB − TMD due to presumptively sustained high levels of MMA associated with TMD.

## 2 Methods

### 2.1 Dataset

The data consisted of recordings from 53 participants with possible SB (i.e., self‐reported SB occurring at least one night per week) collected in previous studies (Miettinen, Myllymaa, Westeren‐Punnonen, et al. 2018; Miettinen, Myllymaa, Hukkanen, et al. 2018). The participants were recruited either with an open call for volunteers, posted on the intranets of the local hospital and university, or by dentists working at dental clinics in the city of Kuopio (Kuopio, Finland) and the Unit of Specialized Oral Care in the Metropolitan Area and Kirkkonummi (Helsinki, Finland). We excluded five participants due to signals containing artefacts or sleep stages that were not scored and four participants because they did not show any MMA events during sleep. If there was more than one night recorded for a participant, only the first night's recording was included in the study. Table 1 presents the demographic information of the included participants.

**TABLE 1:** Number, sex, and age of the included participants as well as their total sleep times.

	Number of participants	Male (%)	Age (years)	Total sleep time (hours)
Total	44	11.4	38.1 ± 10.5	4.8 ± 1.2
No TMD pain group (SB – TMD)	26	15.4	37.3 ± 9.4	5.1 ± 1.3
TMD pain group (SB + TMD)	18	5.6	40.4 ± 12.4	4.5 ± 1.1

*Note:* The values for continuous parameters are presented as mean ± standard deviation.

Abbreviations: SB, possible sleep bruxism; TMD, temporomandibular disorder.

The overnight recordings included EMG of the masseter and submentalis muscles, electroencephalography (Af8‐T9, Af7‐T10, Fp1‐T10, Fp2‐T9), electrooculography, and audio signals. The recordings were conducted at home using an electrode set developed by our group (Miettinen, Myllymaa, Westeren‐Punnonen, et al. 2018; Miettinen, Myllymaa, Hukkanen, et al. 2018) connected to a Nox A1 device (Nox Medical, Reykjavik, Iceland). The participants filled in a questionnaire about TMD (diagnostic criteria for temporomandibular disorders (DC/TMD), Symptom Questionnaire (Ohrbach 2013; Ohrbach et al. 2013)). Before the data collection, the Research Ethics Committee of the Hospital District of Northern Savo, Kuopio, Finland provided a positive statement for the study (34/2013), the National Supervisory Authority for Welfare and Health gave permission for the study (220/2013), and all participants gave written informed consent.

We categorised the participants into two groups based on the DC/TMD questionnaire. The SB + TMD group consisted of participants who reported headache and pain in the jaw, temple, ear, or in front of the ear on either side during the last 30 days, along with changes in headache and pain after opening the mouth or engaging in other jaw activities. The other participants were categorised into the SB − TMD group.

### 2.2 Data Analysis

The bruxism events in left and right masseter EMG signals and sleep stages were scored using Noxturnal Research software (Nox Medical, Reykjavik, Iceland) according to the scoring rules in the American Academy of Sleep Medicine (AASM) Manual version 2 (Berry et al. 2012). More detailed information about the scoring process is provided in the previous publications (Miettinen, Myllymaa, Westeren‐Punnonen, et al. 2018; Miettinen, Myllymaa, Hukkanen, et al. 2018). The EMG signal was filtered with a 10‐Hz high‐pass filter, and MMA events that started during epochs scored as wake were excluded. Notch filtering (50 Hz) was tested but not applied in the final analysis as it did not significantly change the results. The MMA index was determined as the number of MMA events per hour of sleep, and the bruxism time index (BTI) as the percentage of cumulative MMA duration with respect to the total sleep time. The power spectral densities (PSDs) were computed using the Fast Fourier Transform (Cooley and Tukey 1965) of the EMG signals during the MMA events. As the MMA event durations differed, the frequency resolutions varied, and therefore, the resolutions were reduced to the lowest with linear interpolation to obtain equal resolutions. Finally, the absolute power was calculated using the trapezoidal method, and the median frequencies for each PSD were computed. The outliers were identified in PSD parameters as values more than 1.5 times the interquartile range away from 25th and 75th percentiles. The percentage of outliers varied between 0% and 13% depending on the type of MMA event and the calculated parameter.

### 2.3 Statistical Analysis

The statistical significance of differences between the SB + TMD and SB − TMD groups was investigated in terms of absolute power, median signal frequencies, MMA index, BTI, the number of events, the percentage of tonic, phasic, and mixed events, and cumulative event durations. As the PSD‐based parameters included a value for each MMA event, the observations within the groups were not independent and therefore, generalised estimating equations (GEE) test with gamma distribution was used. The GEE is an extension of the generalised model and allows modelling of correlated observations. For other parameters, the Mann–Whitney *U* (MWU) test was utilised. We considered a *p* value of 0.05 as the limit of statistical significance; however, Bonferroni correction for multiple comparisons resulted in an adjusted significance level of 0.002. We used MATLAB (R2022b, The MathWorks, Natick, MA) for data analysis and SPSS (v29; IBM Corp., Armonk, New York) for statistical tests.

## 3 Results

**FIGURE 1:**
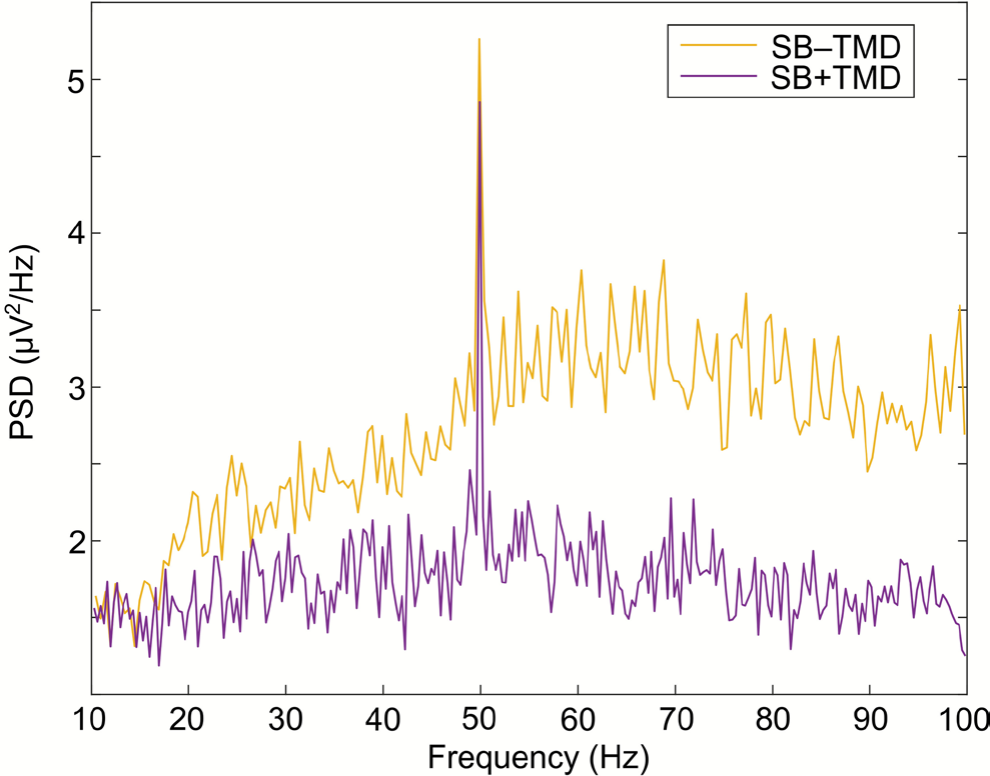
Power spectral densities. Median power spectral densities (PSDs) of the electromyography (EMG) signals during masticatory muscle activation events for possible sleep bruxism (SB) with temporomandibular disorder (TMD) pain (SB + TMD) and without TMD pain (SB − TMD). The high peaks at 50 Hz are due to power line interference in the EMG signals.

Phasic events were the most frequent while mixed events were the least frequent MMA events (Table 2). The percentage of tonic events was lower and that of phasic events higher in the SB + TMD group compared to the SB − TMD group, but the differences were not statistically significant (*p* = 0.737 and *p* = 0.450, respectively). The absolute power (*p* < 0.001) and the median EMG frequencies (*p* = 0.003) were lower in the SB + TMD group than in the SB − TMD group when considering all MMA events (Table 3 and Figure 1). If only tonic MMA events were considered, the median EMG frequencies were different between groups (*p* = 0.004, Table 3), whereas for phasic events, only the absolute power showed a significant difference between groups (*p* < 0.001). The MMA index (*p* = 0.489), BTI (*p* = 0.283), and cumulative event durations (*p* = 0.118) did not differ between the groups.

**TABLE 2:** Total number and percentage of MMA events for the SB + TMD and SB − TMD groups.

	All participants	SB + TMD group	SB − TMD group	*p‐*value (MWU)
All MMA events	396	155	241	0.213
Tonic events	142 (36%)	42 (27%)	100 (41%)	0.147
Phasic events	203 (51%)	101 (65%)	102 (42%)	0.691
Mixed events	51 (13%)	12 (8%)	39 (16%)	0.057

*Note:* The *p* value is shown for the difference between the number of events between SB + TMD and SB − TMD groups.

Abbreviations: MMA, masticatory muscle activity; MWU, the Mann–Whitney *U* test; SB − TMD, possible sleep bruxism without temporomandibular disorder pain; SB + TMD, possible sleep bruxism with temporomandibular disorder pain.

**TABLE 3:** Medians (and interquartile ranges) of MMA events.

	All MMA events	Tonic events	Phasic events	Mixed events
SB + TMD group				
Median frequency (Hz)	55.33 (50.57–59.27)*	54.77 (49.93–59.29)*	55.39 (51.53–58.41)	54.74 (51.06–61.30)
Absolute power (μV^2^)	180.01 (119.14–306.51)**	139.06 (94.02–324.15)	173.17 (135.87–247.00)**	248.45 (172.63–393.05)*
MMA index (1/h)	1.30 (0.70–2.08)	0.43 (0.18–0.74)	0.65 (0.17–1.26)	0.00 (0.00–0.00)
BTI (%)	0.13 (0.06–0.27)	0.03 (0.01–0.06)	0.06 (0.01–0.14)	0.00 (0.00–0.00)
Total event duration (s)	15.57 (9.24–57.66)	3.66 (1.97–9.24)	10.51 (2.80–19.86)	0.00 (0.00–0.00)
SB − TMD group				
Median frequency (Hz)	60.23 (56.61–64.32)	61.24 (56.56–65.00)	58.66 (53.87–62.22)	62.19 (57.62–66.17)
Absolute power (μV^2^)	299.79 (166.66–605.59)	265.63 (155.95–446.10)	276.01 (149.82–591.04)	352.21 (207.19–619.25)
MMA index (1/h)	1.35 (0.86–2.82)	0.73 (0.16–1.09)	0.67 (0.25–1.01)	0.15 (0.00–0.51)
BTI (%)	0.16 (0.10–0.40)	0.06 (0.01–0.10)	0.08 (0.03–0.12)	0.03 (0.00–0.12)
Total event duration (s)	31.18 (20.78–59.36)	8.14 (2.44–17.53)	15.04 (5.29–24.49)	6.31 (0.00–20.20)

*Note:* Outliers are excluded from the frequency‐based values.

Abbreviations: BTI, bruxism time index; MMA, masticatory muscle activity; SB − TMD, possible sleep bruxism without temporomandibular disorder pain; SB + TMD, possible sleep bruxism with temporomandibular disorder pain.

* Statistically significant difference to the SB − TMD group (*p* value < 0.05, the generalised estimating equations test).

** Statistically significant difference to the SB − TMD group (Bonferroni corrected *p* value < 0.002, the generalised estimating equations test).

## 4 Discussion

In this study, we investigated whether nocturnal, MMA event‐associated EMG frequency spectra are related to TMD pain in participants with possible SB. As hypothesised, the absolute power of EMG signal frequencies was significantly lower in the SB + TMD group compared to the SB − TMD group (*p* < 0.001), indicating greater muscle fatigue in SB + TMD participants. However, the median frequencies indicated only a trend of this association when corrected for multiple comparisons (*p* = 0.003). On the other hand, the traditional metrics, that is, MMA index and BTI, showed no significant differences between the groups. To the best of our knowledge, no previous studies have investigated the association between the EMG frequency spectra and TMD consequences in SB participants. The results are in line with the pain adaptation model, which suggests that muscle activation is decreased due to pain (Lund et al. 1991).

Only few studies have performed a frequency‐domain analysis of MMA events, but these studies did not investigate its association with the consequences of SB. Instead, these studies typically compared SB participants to controls or focused on developing algorithms for the detection of MMA events or the assessment of SB (Bin Heyat et al. 2020; Farella et al. 2009; Kato et al. 2003; Lai et al. 2019; Sonmezocak and Kurt 2021). These results are also conflicting—one study did not find differences in the absolute power of EMG signals between SB and control participants (Kato et al. 2003) whereas another study found that the average normalised values of PSD during wake and rapid‐eye movement sleep were higher in SB compared to control subjects (Lai et al. 2019). Furthermore, de Paula Gomez et al. calculated the median frequency of the power spectrum during voluntary clenching to investigate the effect of different treatment modalities for SB + TMD but found no significant differences between pre‐ and post‐treatment (de Paula Gomes et al. 2014). Thus, research on characteristics of the EMG power spectrum in SB participants is limited and results contradictory.

Frequency spectrum analysis has, however, been conducted in TMD patients without reported bruxism during a sustained clenching task while awake. Our results are in line with previous studies which have reported lower EMG frequencies in TMD patients than in controls (Castroflorio et al. 2012; Gay et al. 1994; Ries et al. 2016; Tartaglia et al. 2011) whereas other studies reported differences only in the rate of power frequency shift during sustained contractions (Koyano et al. 1995; Kroon and Naeije 1992; Liu et al. 1999; Park et al. 2012; Pitta et al. 2015; Woźniak et al. 2015). However, some studies reported higher EMG frequencies in TMD patients compared to controls (Lauriti et al. 2013; Pires and Rodrigues‐Bigaton 2018; Politti et al. 2016) while others found no differences between groups (Rudy et al. 2001; Xu et al. 2017) or reported mixed results (Hori et al. 1995). These contradictory results can be due to different muscles being monitored, different subtypes of TMD investigated (e.g., arthrogenous or myogenous TMD), or differences in the clenching tasks. In any case, it is possible that the lower EMG frequencies in our study are in fact due to the presence of TMD while SB has no effect. However, the results of the present study indicate that the effect is also seen during sleep, which has not, to the best of our knowledge, been reported before.

The results of phasic MMA events were more similar between the SB + TMD and SB − TMD groups compared to tonic events. This could suggest that tonic events are more closely associated with TMD compared to phasic events. However, the low number of tonic events in the SB + TMD group might also explain this result and decrease the power of statistical analysis. However, this could be a typical phenomenon in SB as Rossetti et al. also reported a significantly lower percentage of tonic events in SB participants with myofascial pain compared to SB participants without myofascial pain (Rossetti, de Araujo, et al. 2008). In addition, other studies have found that tonic events are significantly longer in SB participants with morning masticatory muscle fatigue or tenderness compared to controls (Yoshida et al. 2017) and that sustained low‐level tonic events are more prevalent and longer in subjects with a history of orofacial pain compared to controls during wakefulness but not during sleep (Mude et al. 2018). This further indicates that tonic events seem to be associated with the presence of TMD. However, we found no significant differences in the number or cumulative duration of events between groups. The low number of mixed events suggests that future studies with larger sample sizes are needed to confirm the association between TMD and different MMA event types.

The results of our study indicate higher muscle fatigue in the SB + TMD group compared to the SB − TMD group. However, we did not conduct muscle fatigue testing and cannot draw definite conclusions about the origin of this possible muscle fatigue. It could be due to SB, awake bruxism, or other factors not associated with SB. It is possible that TMD alone may lead to altered muscle recruitment, affecting EMG frequency, irrespective of SB. We did not include a control group of participants without SB because MMA events are not frequently observed in those individuals, making meaningful comparison difficult. As the MMA index or BTI did not differ between groups, it seems that the number or duration of MMA events during the monitored night was not associated with the fatigue or TMD consequences. However, the MMA indices were relatively low, and we did not evaluate the magnitude of the MMA events, which might affect muscle fatigue and TMD pain. This was because maximum voluntary contraction measurements were not conducted, and therefore, we could not normalise the EMG amplitudes. However, the electrode set was similar for each participant, and the signals were visually checked for artefacts during the scoring process to mitigate the possible effects of variations in electrode placements and poor signal quality on the results. In addition, the relationship between MMA and TMD might not be linear (Svensson et al. 2008; Manfredini et al. 2019). It is possible that TMD could have originated from intense MMA during previous nights, and the resulting pain lowers the levels of MMA on the following nights; however, low levels of MMA still maintain muscle fatigue and pain (Manfredini et al. 2019). This hypothesis needs, however, to be investigated in the future with long‐term EMG monitoring.

The frequency‐domain analysis conducted in this study may be useful in determining the pathophysiology of TMD or SB. This methodology might reveal aspects of the history of these conditions, such as a high level of muscle activation before the recording, while the traditional MMA index and BTI give information on the number and duration of MMA events during a single night only. The frequency‐domain analysis could also be useful in monitoring the effectiveness of treatment. However, this needs to be confirmed in future studies, as de Paula Gomez et al. reported no significant differences between pre‐ and post‐treatment EMG median frequencies during voluntary clenching, even though improvement in TMD was seen (de Paula Gomes et al. 2014). Furthermore, as muscle fatigue is a possible negative outcome of bruxism, adding fatigue analysis in current research and clinical protocols would be useful.

The strength of this study is the extensive home measurement set‐up that included electroencephalography and sleep staging. The success rate and technical quality of the electrode set have been evaluated as comparable to regular type II home polysomnography (Miettinen, Myllymaa, Westeren‐Punnonen, et al. 2018). Moreover, findings from our previous validation study (Miettinen, Myllymaa, Muraja‐Murro, et al. 2018) demonstrated that the electrode set enables reliable classification of individuals as bruxers or non‐bruxers, with results consistent with those obtained using standard in‐lab (type I) polysomnography. Therefore, we do not expect that the results would substantially differ with type I polysomnography. However, due to the small and unintentionally imbalanced sample size between male and female participants, along with only 18 participants in the SB + TMD group, the statistical power is limited (approximately 0.7). Therefore, the results should be validated in future studies involving a larger and more heterogeneous pool of participants. Moreover, we included participants with possible (i.e., self‐reported) SB, and the MMA levels and total sleep times were relatively low in this group of participants, which could be due to night‐to‐night variability or first‐night effect. The night‐to‐night variability in the SB activity could lead to misinterpretation of the relationship between SB and TMD. This is a well‐known limitation in sleep research and clinical practice, as typically only a single‐night recording is conducted. In this study, a subset of participants (*N* = 17) had recordings from three consecutive nights, and we reanalysed the data using those instead of the first night's recording. Similarly to the first night, the absolute power and the median frequency differed between the SB + TMD and SB − TMD groups for the second (both *p* < 0.002) and the third night (*p* < 0.002 and *p* < 0.05, respectively), and thus, the night‐to‐night variability is unlikely to affect the results. In addition, we assessed the TMD symptoms with a questionnaire, whereas clinical examination could have provided a more accurate estimation of the current pain status, as self‐reported pain may vary across days, leading to misclassification. Therefore, the self‐reports from the last 30 days may not necessarily reflect the night at which the measurements were conducted, and thus, the results of the current study need to be confirmed in a study where the TMD diagnosis, pain status, and pain intensity are clinically evaluated right before and after the EMG measurement. Other limitations are the absence of a distinction between arthrogenous and myogenous TMD pain, as well as the lack of EMG amplitude analysis and muscle fatigue testing. Moreover, we did not assess potential confounding factors such as anxiety, medication use, sleep quality, differences in the sleep physiology, muscle tone, sex, neurological or psychiatric diseases, structural abnormalities in the brain, or comorbid pain conditions, which could have affected the results. Lastly, the sampling rate of EMG was relatively low (200 Hz), limiting the frequency analysis to 100 Hz, which could lead to erroneous interpretation of the relationship between EMG signal frequencies and SB. Therefore, it would have been beneficial to collect data with a device achieving higher resolution, as previous studies have found EMG activation also at frequencies above 100 Hz (Naeije and Zorn 1981). However, the median frequencies observed in this study were approximately 50–60 Hz. These values fall well within the expected range for surface EMG signals and suggest that the current measurement technique does not introduce significant bias. Due to these limitations, this study should be regarded more as an exploratory pilot study presenting preliminary findings rather than a confirmatory study.

## 5 Conclusion

In conclusion, the EMG frequency spectrum calculated during MMA events might serve as an indicator of SB severity related to TMD pain and may be useful in determining the pathophysiology of TMD but this needs to be validated in future studies. The lower EMG frequencies observed in the SB + TMD participants suggest muscle fatigue, possibly due to the intense bruxism activity in the past. On the contrary, the conventional MMA index and BTI did not differ between the groups, indicating that they are poor markers of TMD. The study also questioned the usefulness of traditional parameters in assessing the severity of SB, suggesting that new approaches are urgently needed.

## Author Contributions

**Minna Pitkänen:** conceptualization, formal analysis, investigation, methodology, software, funding acquisition, visualization, writing – original draft, writing – review and editing. **Miro Rytkönen:** methodology, software, writing – review and editing. **Tomi Miettinen:** methodology, data curation, writing – review and editing. **Jari Ahlberg:** writing – review and editing. **Frank Lobbezoo:** writing – review and editing. **Katja Myllymaa:** methodology, data curation, writing – review and editing. **Juha Töyräs:** funding acquisition, writing – review and editing. **Timo Leppänen:** funding acquisition, writing – review and editing. **Sami Myllymaa:** methodology, data curation, funding acquisition, writing – review and editing.

## Funding

This work was supported by the State Research Funding for University‐Level Health Research, Kuopio University Hospital, Wellbeing Services County of North Savo (50TP012, 5041797, 5041803, 5041820), Selma and Maja‐Lisa Selander's Fund (Minerva Foundation), Research Foundation of the Pulmonary Diseases, Sigrid Jusélius Foundation, Foundation of the Finnish Anti‐Tuberculosis Association, Research Council of Finland (361199).

## Ethics Statement

The Research Ethics Committee of the Hospital District of Northern Savo, Kuopio, Finland, provided a positive statement (34/2013) and the National Supervisory Authority for Welfare and Health gave permission for the study (220/2013).

## Conflicts of Interest

F.L. receives research grants from Health Holland and the Dutch Research Council (NWO), unrelated to this study.

## Data Availability

The data underlying this article cannot be shared publicly due to the privacy of individuals that participated in the study.
